# Activated pathogenic Th17 lymphocytes induce hypertension following high-fructose intake in Dahl salt-sensitive but not Dahl salt-resistant rats

**DOI:** 10.1242/dmm.044107

**Published:** 2020-05-27

**Authors:** Eunjo Lee, Namkyung Kim, Jinjoo Kang, Sangwon Yoon, Hae-Ahm Lee, Hanna Jung, Sang-Hyun Kim, Inkyeom Kim

**Affiliations:** 1 Department of Pharmacology, School of Medicine, Kyungpook National University, Daegu 41944, Republic of Korea; 2 Cardiovascular Research Institute, Kyungpook National University, Daegu 41944, Republic of Korea; 3 BK21 Plus KNU Biomedical Convergence Program, Department of Biomedical Science, School of Medicine, Kyungpook National University, Daegu 41944, Republic of Korea; 4 Medical Research Center for Bioreaction to Reactive Oxygen Species and Biomedical Science Institute, School of Medicine, Graduate School, Kyung Hee University, Seoul 02447, Republic of Korea; 5 Department of Thoracic and Cardiovascular Surgery, Kyungpook National University Hospital, Daegu 41944, Republic of Korea

**Keywords:** Th17 lymphocytes, Treg lymphocytes, Fructose, Hypertension, Dahl salt-sensitive rat, Dahl salt-resistant rat

## Abstract

High-salt intake and high-fructose intake are risk factors for hypertension via oxidative stress and inflammation. T helper (Th)17 lymphocytes play an important role in the development of hypertension. Here, we tested the hypothesis that activation of pathogenic Th17 lymphocytes induces hypertension after high-fructose intake in Dahl salt-sensitive (SS) but not Dahl salt-resistant (SR) rats. Eight-week-old male SS and SR rats were offered 20% fructose solution or tap water only for 4 weeks. Systolic blood pressure was measured by the tail-cuff method. T lymphocyte [Th17 and T regulatory (Treg)] profiling was determined via flow cytometry. The expression of Th17-related (IL-17A, IL-17RA, IL-23R and RORγt) and Treg-related (IL-10, CD25, FOXP3 and TGFβ) factors were measured via ELISA or qRT-PCR. Th17 lymphocytes isolated from high-fructose-fed SS rats were intraperitoneally injected into recipient SS and SR rats, and recombinant IL-23 protein was subcutaneously injected into SS and SR rats to induce hypertension.

High-fructose intake induced hypertension via the activation of pathogenic Th17 lymphocytes in SS but not SR rats. Injection of activated Th17 lymphocytes isolated from fructose-fed SS rats induced hypertension via increase of serum IL-17A only in recipient SS rats. In addition, injection of IL-23 induced hypertension via activation of pathogenic Th17 lymphocytes only in SS rats.

Thus, activation of pathogenic Th17 lymphocytes induces hypertension after high-fructose intake in SS but not SR rats. These results indicate that immunologic tolerance plays an important role in protection against hypertension in SR rats.

## Abstract (precis)

**Summary:** Activation of pathogenic Th17 lymphocytes induces hypertension after high-fructose intake in salt-sensitive rats. Immune activation plays an important role in the development of hypertension, whereas immune tolerance is protective against hypertension.

## INTRODUCTION

Hypertension is a major risk factor for stroke, cardiovascular disease and renal disease, and a big burden to the total annual health budget worldwide. In the near future, the number of people with hypertension is expected to reach 1.56 billion (Ha et al., 2012; Kearney et al., 2005). Although some cases of hypertension are a result of clear causes, such as renal problems, abnormal cardiac output, excessive adrenal aldosterone production or genetic causes, more than 90% of cases do not have an identifiable etiology and are classified as ‘essential’. Essential hypertension often coexists with obesity, abnormal lipid metabolism, aging and insulin resistance, and thus is often considered as part of a complicated metabolic phenotype (Carretero and Oparil, 2000).

High-fructose corn syrup and sucrose are added to various food products, and fructose-sweetened beverages are a major source of dietary fructose intake worldwide (Hannou et al., 2018). The continuous rise in fructose intake has been reflected in the increase in the prevalence of cardiometabolic syndrome, including hypertension, over the past century (Johnson et al., 2007). Dietary components that increase blood pressure have become a big issue, and many research groups have recently started to pay attention to fructose intake (Taskinen et al., 2019). Fructose elevates the blood pressure through oxidative stress, which induces hemodynamic effects, such as increased sympathetic nervous system activity, endothelial dysfunction, activation of the renin-angiotensin-aldosterone system and inflammation (Caliceti et al., 2017; Johnson et al., 2009). Furthermore, high-fructose, but not high-glucose, intake with a high-salt diet induces salt-sensitive hypertension through reduced sodium excretion, increased salt retention and impaired renal nitric oxide excretion (Ares and Ortiz, 2015; Gordish et al., 2017).

The immune system plays a prominent role in each stage of hypertension and contributes to cardiovascular pathology, end-organ damage and mortality. Studies have shown coherent relationships between hypertension, pro-inflammatory cytokines and certain cell types in the immune systems (Singh et al., 2014). CD4^+^ T cells, effector T lymphocytes that include T helper (Th)1, Th2 and Th17 subsets of lymphocytes, play significant roles in hypertension and other cardiovascular disease (Norlander et al., 2018). A particularly important cytokine in the development of hypertension is interleukin (IL)-17, which is mainly produced by Th17 cells that have a representative master transcription factor, retinoic acid receptor-related orphan receptor (ROR)γt. IL-23 plays a crucial role in stabilizing and reinforcing the Th17 phenotype by increasing expression of IL-23 receptor (IL-23R) and endowing Th17 cells with pathogenic functions (Wu et al., 2013; Zhou et al., 2007). T regulatory (Treg) lymphocytes are involved in self-tolerance and maintain immune homeostasis. The anti-inflammatory cytokine IL-10 is a key product of Treg cells, which have a representative master transcription factor, forkhead box P3 (FOXP3) (Ouyang et al., 2010). *Il-10*^−/−^ mice exhibit enhanced hypertension, endothelial dysfunction and increased oxidative stress in response to hypertensive stimuli (Barhoumi et al., 2011).

It has been shown that imbalance between Th17 and Treg cells is important for inducing cardiovascular diseases including hypertension in several hypertensive animal models (Katsuki et al., 2015). Serum/glucocorticoid-regulated kinase 1 (SGK1) is a critical factor that reciprocally regulates development of Th17/Treg balance (Wu et al., 2018). SGK1 regulates the development and function of Th17 cells and has inhibitory effects on Treg cells. IL-23R acts upstream of SGK1 signaling. SGK1 also plays a critical role in IL-23R-mediated inhibition of Treg cells and pathologic activation of Th17 cells through phosphorylation of transcription factor forkhead box O1/3 (FOXO1/3) in experimental models (Binger et al., 2015). Phosphorylated FOXO1/3 translocate from the nucleus to the cytosol during Th17/Treg differentiation. FOXO1/3 exist in the nucleus of Th17 cells, attenuating the development of Th17 cells and transcription of their related genes (Lainé et al., 2015). FOXO1/3 also exist in the nuclei of Treg cells, stabilizing FOXP3 and subsequently increasing the transcriptional activity of Treg cell-related genes (Hernandez et al., 2015).

Dahl salt-sensitive (SS) and Dahl salt-resistant (SR) rats, with the latter showing normotensive control, are inbred rat models widely used to study salt-sensitive hypertension. A high-salt diet induces hypertension in SS rats but rarely affects the blood pressure of SR rats (Bashyam, 2007). Previous studies have shown that *Rag1*^−/−^ and *Sh2b3*^−/−^ (*Sh2b3* plays a suppressive role in the activation of immune responses and cytokine signaling) SS rats were protected from hypertension after having been fed a high-salt diet (Mattson et al., 2013; Rudemiller et al., 2015). Most recently, SS rats of both sexes fed other diets, such as a high-fat diet, have been shown to develop increased blood pressure through a T cell profile change (Taylor et al., 2018). Taken together, these studies suggest that the immune system plays a critical role in inducing hypertension in SS rats. However, SR rats have not been the focus in most studies. In addition, the effects of high-fructose intake on physiological changes in SS and SR rats, and underlying molecular mechanisms, are not fully understood. In this study, we tested the hypothesis that activation of pathogenic Th17 lymphocytes induces hypertension after high-fructose intake in SS but not SR rats.

## RESULTS

### High-fructose intake induces hypertension and glucose intolerance in SS but not SR rats

**Fig. 1.**
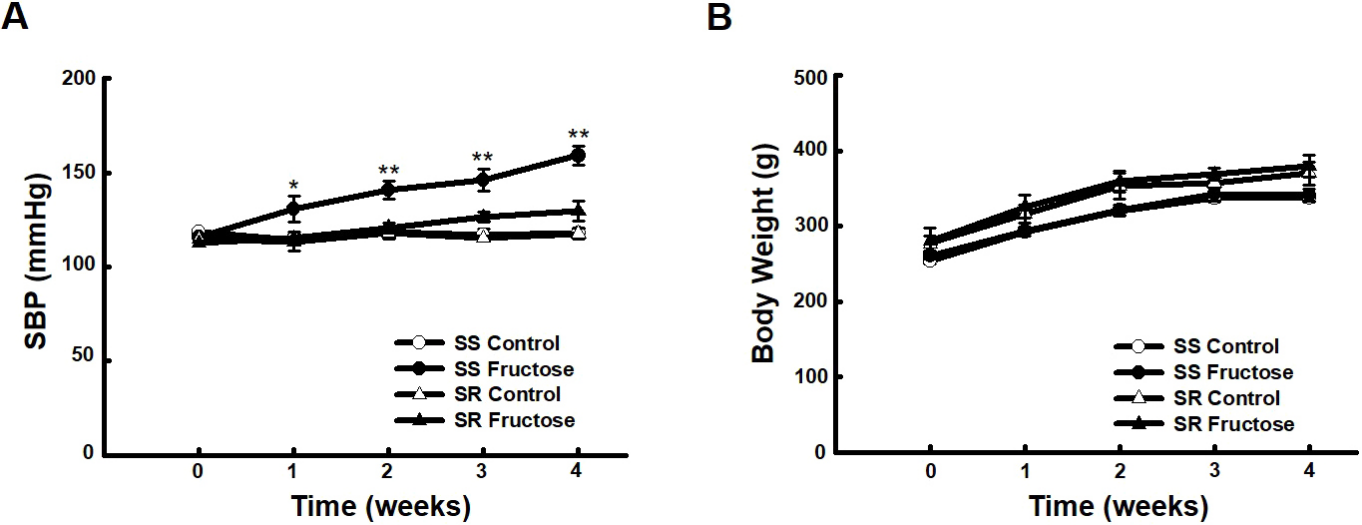
**Effects of high-fructose intake on systolic blood pressure (SBP) and body weight of Dahl salt-sensitive (SS) and Dahl salt-resistant (SR) rats.** (A) SBP was measured using the tail-cuff method in SS and SR rats offered 20% fructose in drinking water or water only for 4 weeks. High-fructose intake induced hypertension in SS but not SR rats. [Kruskal–Wallis test and one-way ANOVA followed by Tukey's HSD post hoc tests were applied to compare multiple data. Data shown are the mean±s.e.m. of six independent experiments (*P<0.05 and **P<0.01 versus the SS rat control group).] (B) The body weights of the rats were monitored weekly for 4 weeks and were unaffected by high-fructose intake.

To determine whether high-fructose intake had an effect on blood pressure, we measured the systolic blood pressure (SBP) of the rats for 4 weeks. High-fructose intake significantly increased the blood pressure of SS but not SR rats (Fig. 1A), without affecting the body weight in either group (Fig. 1B). To confirm whether high-fructose intake induces glucose intolerance in SS and SR rats, we performed a glucose tolerance test (GTT). High-fructose intake caused glucose intolerance in SS but not SR rats (Fig. S1). Furthermore, to determine whether high-fructose intake affects vascular relaxation, we conducted an organ bath study. There was no difference in vascular relaxation responses among all groups (Fig. S2).

### High-fructose intake increases Th17 cells and their cytokine activity in SS but not SR rats

**Fig. 2.**
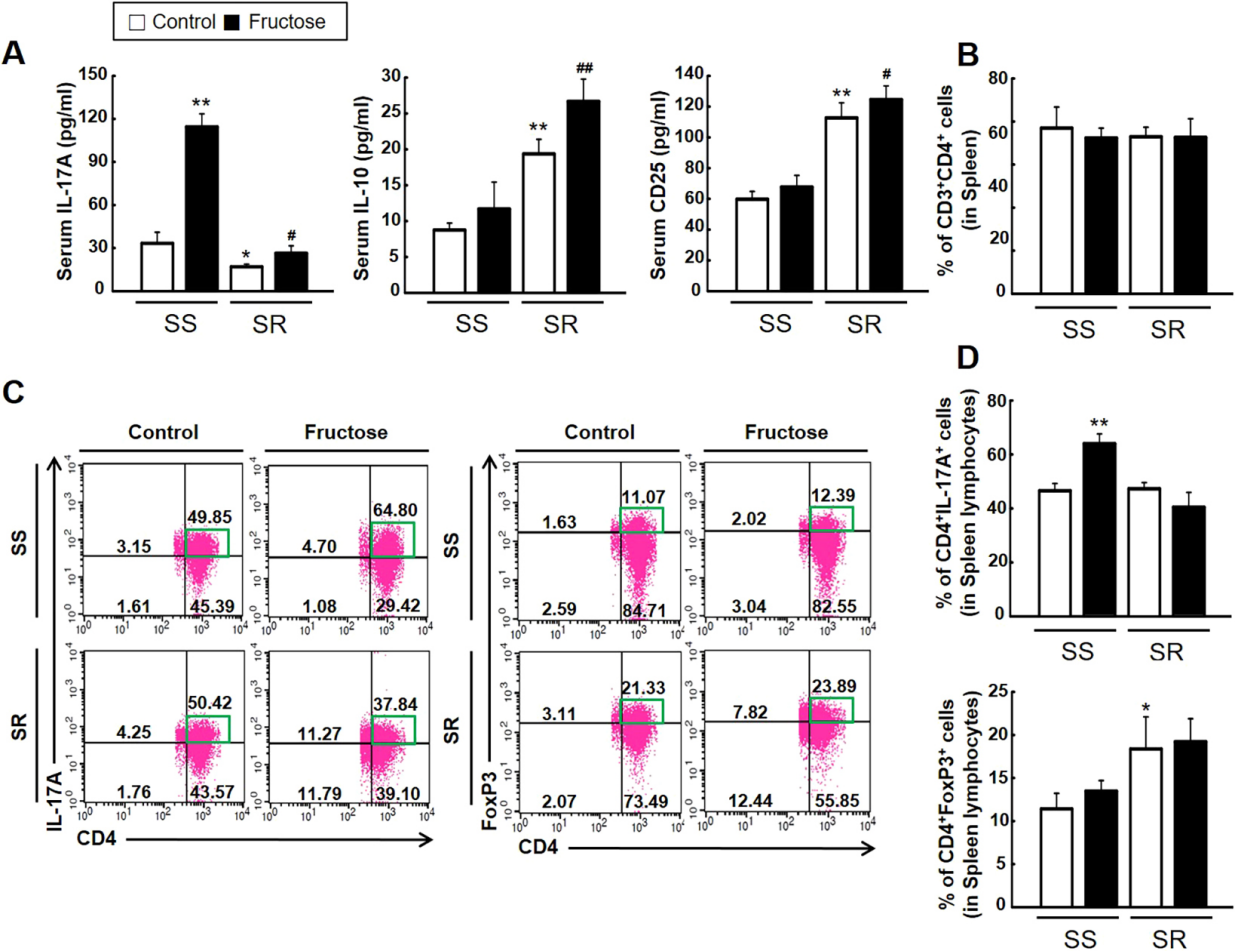
**Effects of high-fructose intake on blood cytokines and T cell profiles in SS and SR rats.** (A) The serum levels of a pro-inflammatory cytokine (IL-17A), an anti-inflammatory cytokine (IL-10) and a secretory cell surface marker (CD25) were detected via ELISA in SS and SR rats offered 20% fructose in drinking water or tap water only for 4 weeks. [Student's *t*-tests were performed for the analysis of significant differences between the two groups. Data shown are the mean±s.e.m. of six independent experiments (*P<0.05 and **P<0.01 versus the SS rat control group; ^#^*P*<0.05 and ^##^*P*<0.01 versus the SR rat control group).] High-fructose intake increased the level of IL-17A much more in SS than in SR rats, whereas it increased the level of IL-10 and CD25 in SR but not SS rats. (B) The percentages of total CD3^+^CD4^+^ cells in the spleen in SS and SR rats. (C,D) Representatives (processed by CellQuest) (C) and densitometry (D) of the flow cytometry conducted on Th17 (CD4^+^IL-17A^+^) lymphocytes and Treg (CD4^+^FOXP3^+^) lymphocytes obtained from the splenocytes of SS and SR rats. High-fructose intake elevated the population of Th17 cells in SS but not SR rats. [Student's *t*-tests were performed for the analysis of significant differences between the two groups. Data shown are the mean±s.e.m. of six independent experiments (*P<0.05 and **P<0.01 versus the SS rat control group).]

**Fig. 3.**
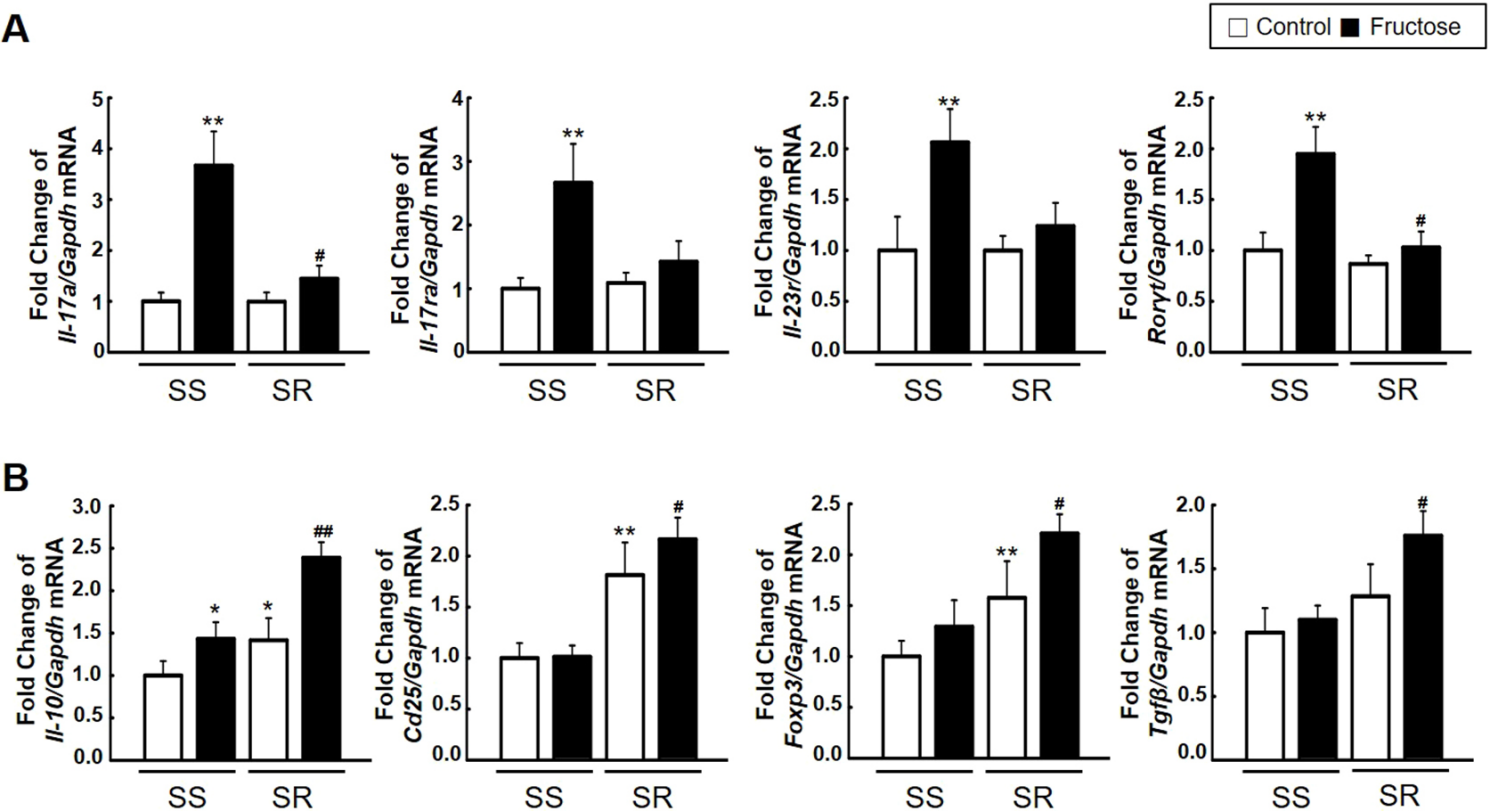
**Effects of high-fructose intake on the expression of Th17 and Treg lymphocyte-related genes in spleens of SS and SR rats.** (A) mRNA expression levels of Th17 lymphocyte-related genes *Il-17a*, *Il-17ra*, *Il-23r* and *RORγt* in the spleens of SS and SR rats were measured by qRT-PCR. [Student's *t*-tests were performed for the analysis of significant differences between the two groups. Data shown are the mean±s.e.m. of six independent experiments (**P<0.01 versus the SS rat control group; ^#^*P*<0.05 versus the SR rat control group).] High-fructose intake increased the mRNA expression of Th17 lymphocyte-related genes more in SS than in SR rats. (B) mRNA expression levels of Treg lymphocyte-related genes *Foxp3*, *Cd25*, *Il-10* and *Tgfβ* were measured by qRT-PCR in the spleens of SS and SR rats. High-fructose intake increased the mRNA expression of Treg lymphocyte-related genes more in SR rats than in SS rats. [Student's *t*-tests were performed for the analysis of significant differences between the two groups. Data shown are the mean±s.e.m. of six independent experiments (*P<0.05 and **P<0.01 versus the SS rat control group; ^#^*P*<0.05 and ^##^*P*<0.01 versus the SR rat control group).]

To investigate whether high-fructose intake affects the immune response, we analyzed blood cytokines and the T cell profile in SS and SR rats. Enzyme-linked immunosorbent assay (ELISA) showed that high-fructose intake significantly elevated the serum level of IL-17A (a pro-inflammatory cytokine) much more in SS than in SR rats. SR rats have higher serum levels of IL-10 (an anti-inflammatory cytokine) and CD25 (also known as IL2RA; a surface marker of activated Treg lymphocytes) than SS rats. High-fructose intake further increased the level of IL-10 and CD25 in SR but not SS rats (Fig. 2A). Next, to determine whether high-fructose intake affects T cell profiling among splenocytes, we conducted flow cytometry, the gating strategy for which is shown in Fig. S3. The population of CD4^+^ T cells in the spleen was not different among all groups (Fig. 2B). High-fructose intake increased the population of CD4^+^IL-17A^+^ (Th17) lymphocytes in SS but not SR rats. The population of CD4^+^FOXP3^+^ (Treg) lymphocytes was higher in SR than in SS rats, and was not affected by high-fructose intake in either rat group (Fig. 2C,D). Furthermore, to explore whether high-fructose intake has any effect on the expression of genes related to Th17 and Treg lymphocytes, we analyzed mRNA expression in the spleen using quantitative real-time polymerase chain reaction (qRT-PCR). High-fructose intake significantly increased the mRNA expression of Th17 lymphocyte-related genes *Il-17a*, interleukin 17 receptor A (*Il-17ra*), *Il-23r* and *RORγt* (also known as *Rorc*) much more in SS than in SR rats (Fig. 3A). By contrast, high-fructose intake increased the mRNA expression of Treg lymphocyte-related genes *Foxp3*, *Cd25*, *Il-10* and transforming growth factor beta (*Tgfβ*; also known as *Tgfb1*) much more in SR rats than in SS rats (Fig. 3B). We also investigated the effect of high-fructose intake on gene expression of Th17-related and Treg-related factors in the kidneys of SS and SR rats. These data showed that almost similar renal gene expression patterns were observed in the spleen of SS and SR rats (Fig. S4).

### High-fructose intake increased SGK1 signaling in SS but not SR rats

**Fig. 4.**
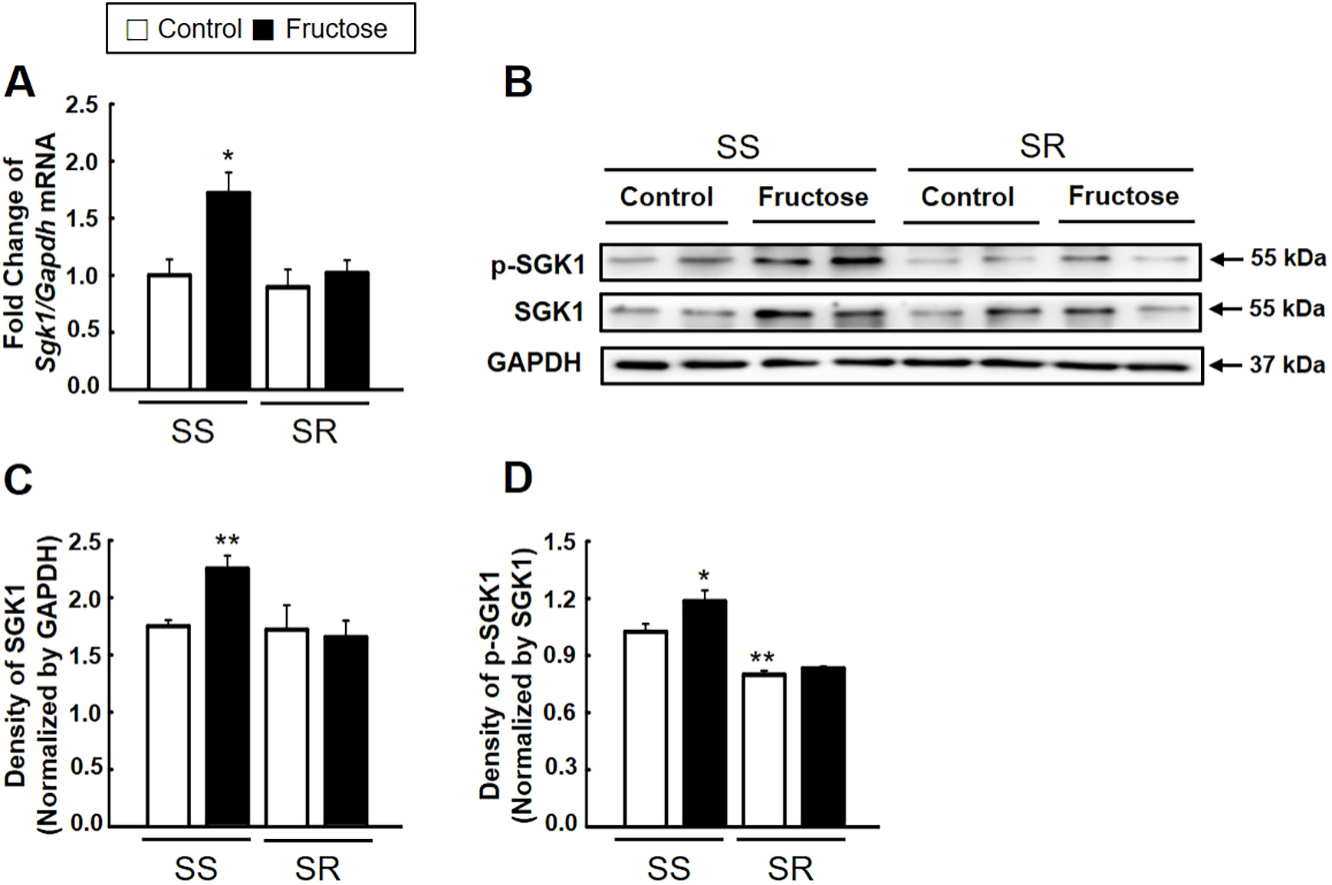
**Effect of high-fructose intake on the expression and activation of SGK1 in SS and SR rats.** (A) The mRNA expression level of *Sgk1* was detected by qRT-PCR in the spleen of SS and SR rats. [Student's *t*-tests were performed for the analysis of significant differences between the two groups. Data shown are the mean±s.e.m. of six independent experiments (*P<0.05 versus the SS rat control group).] (B-D) Representative western blot (B) and densitometry (C,D) of phosphorylated (p)-SGK1 and SGK1 in the spleen of SS and SR rats. High-fructose intake increased the expression and activation of SGK1 in SS but not SR rats. [Student's *t*-tests were performed for the analysis of significant differences between the two groups. Data shown are the mean±s.e.m. of six independent experiments (*P<0.05 and **P<0.01 versus the SS rat control group).]

**Fig. 5.**
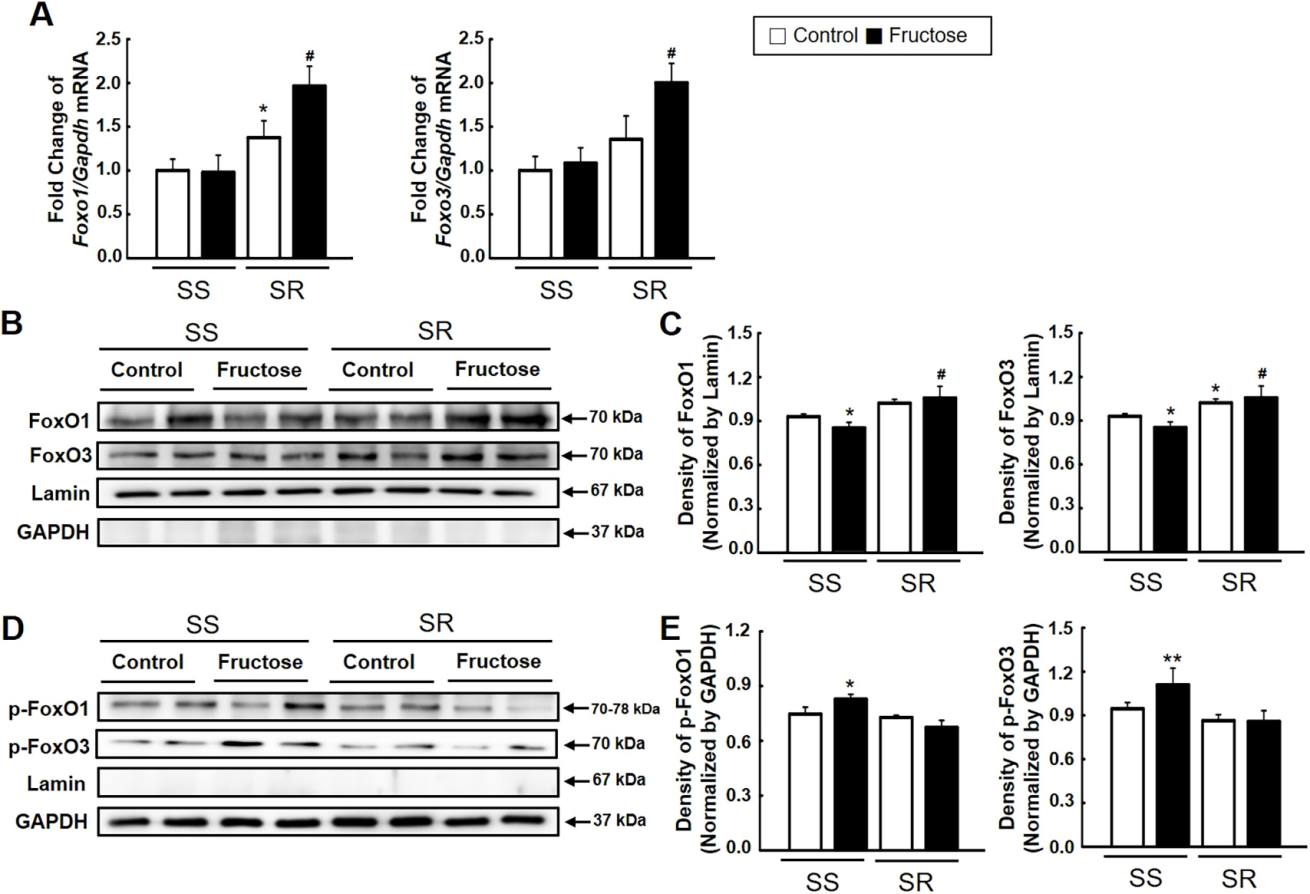
**Effect of high-fructose intake on the expression and phosphorylation of FOXO1/3 in SS and SR rats.** (A) The mRNA expression level of *Foxo1/3* in the spleens of SS and SR rats was detected by qRT-PCR. [Student's *t*-tests were performed for the analysis of significant differences between the two groups. Data shown are the mean±s.e.m. of six independent experiments (*P<0.05 versus the SS rat control group; ^#^*P*<0.05 versus the SR control group).] (B-E) Representatives (B,D) and densitometry (C,E) of the expression of FoxO1/3 and p-FOXO1/3 using western blotting of the nuclear and cytosolic fractions, respectively, in the spleens of SS and SR rats. High-fructose intake increased the expression of FOXO1/3 in SR rats as well as the phosphorylation of FOXO1/3 in SS rats. [Student's *t*-tests were performed for the analysis of significant differences between the two groups. Data shown are the s.e.m. of six independent experiments (*P<0.05 and **P<0.01 versus the SS control group; ^#^*P*<0.05 versus the SR control group).]

We performed qRT-PCR and western blot analysis to determine whether high-fructose intake affects the expression of SGK1 and its activity. High-fructose intake significantly increased the mRNA (Fig. 4A) and protein expression of SGK1 (Fig. 4B,C) in SS but not SR rats. SGK1 activity measured by the phosphorylation level of SGK1 was higher in SS rats than in SR rats, and was further increased by high-fructose intake (Fig. 4B,D). We conducted qRT-PCR and western blot analysis of FOXO1/3 and its phosphorylation level to examine whether high-fructose intake affects its expression. High-fructose intake significantly increased *Foxo1/3* mRNA only in SR rats (Fig. 5A). High-fructose intake decreased the amount of nuclear FOXO1/3 (active form) in SS rats but increased it in SR rats (Fig. 5B,C). However, high-fructose intake increased the amount of cytosolic phospho-FOXO1/3 (p-FOXO1/3; inactive form) in SS rats (Fig. 5D,E).

### Transfer of activated pathogenic Th17 lymphocytes induced hypertension

**Fig. 6.**
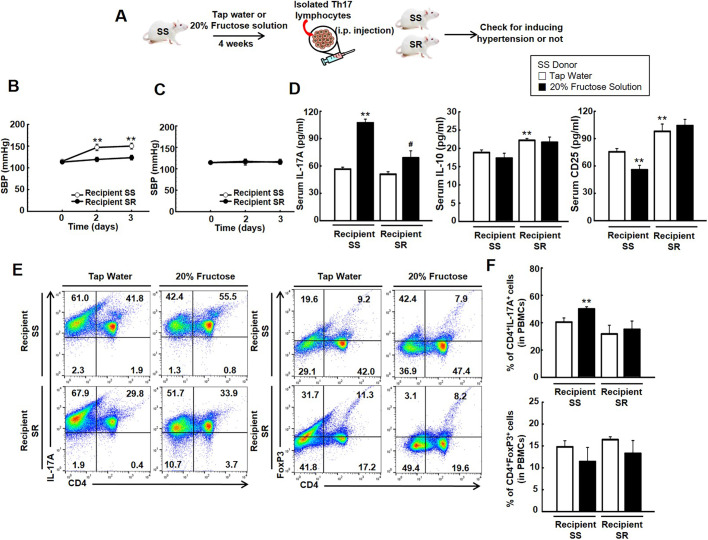
**Effect of transferring activated Th17 lymphocytes on the blood pressure of recipient SS and SR rats.** (A) Schematic of Th17 lymphocyte transfer experiments reported in B-F. (B,C) SBP measurements (using the tail-cuff method) of recipient SS and SR rats injected with Th17 lymphocytes isolated from SS rats on 20% fructose for 4 weeks (B) or Th17 lymphocytes isolated from the tap water group (C). [Student's *t*-tests were performed for the analysis of significant differences between the two groups. Data shown are the mean±s.e.m. of six independent experiments (**P<0.01 versus the SS rat control group).] (D) The levels of serum IL-17A, IL-10 and CD25 were detected via ELISA in recipient SS and SR rats 3 days after injection. Th17 lymphocytes obtained from SS rats on 20% fructose for 4 weeks increased the level of IL-17A profoundly more in recipient SS than in SR rats. [Student's *t*-tests were performed for the analysis of significant differences between the two groups. Data shown are the mean±s.e.m. of six independent experiments (**P<0.01 versus the SS control group; ^#^*P*<0.05 versus the SR control group).] (E,F) Representatives (processed by FlowJo v10.0) (E) and densitometry (F) of the flow cytometry conducted on PBMCs from recipient SS and SR rats 3 days after the injection of Th17 lymphocytes. Injection of Th17 cells (obtained from SS rats maintained on 20% fructose for 4 weeks) elevated the population of Th17 cells in recipient SS but not SR rats. [Student's *t*-tests were performed for the analysis of significant differences between the two groups. Data shown are the mean±s.e.m. of six independent experiments (**P<0.01 versus the SS control group).]

Given our findings that high-fructose intake induced hypertension and increased the population of Th17 lymphocytes in SS rats (Figs S1 and S2, respectively), we investigated whether high-fructose intake induces activated pathogenic Th17 lymphocytes, and also whether these activated pathogenic Th17 lymphocytes induce hypertension. Blood pressure was elevated in recipient SS but not recipient SR rats, which had been injected with Th17 lymphocytes from donor SS rats on 20% fructose solution for 4 weeks (Fig. 6B). However, there was little blood pressure change in recipient SS and SR rats injected with Th17 lymphocytes isolated from SS rats on tap water (Fig. 6C). We conducted ELISA to examine whether the injected Th17 lymphocytes contribute to the levels of blood cytokines and secretory cell surface marker CD25. The transfer of Th17 lymphocytes (from donor SS rats on 20% fructose solution for 4 weeks) significantly elevated the level of serum IL-17A much more in recipient SS than in recipient SR rats, with little change in the level of serum IL-10 in either group. The transfer of Th17 lymphocytes (from donor SS rats on 20% fructose solution for 4 weeks) decreased the level of serum CD25 in recipient SS but not recipient SR rats (Fig. 6D). We performed flow cytometry analysis to investigate whether the transfer of Th17 lymphocytes affects T cell profiles in peripheral blood mononuclear cells (PBMCs). The transfer of Th17 lymphocytes (from donor SS rats on 20% fructose solution for 4 weeks) increased the population of CD4^+^IL-17A^+^ (Th17) cells in recipient SS but not recipient SR rats, with little change in the population of CD4^+^FOXP3^+^ (Treg) cells in either group (Fig. 6E,F).

### IL-23 plays a role in inducing hypertension through activation of pathogenic Th17 lymphocytes

**Fig. 7.**
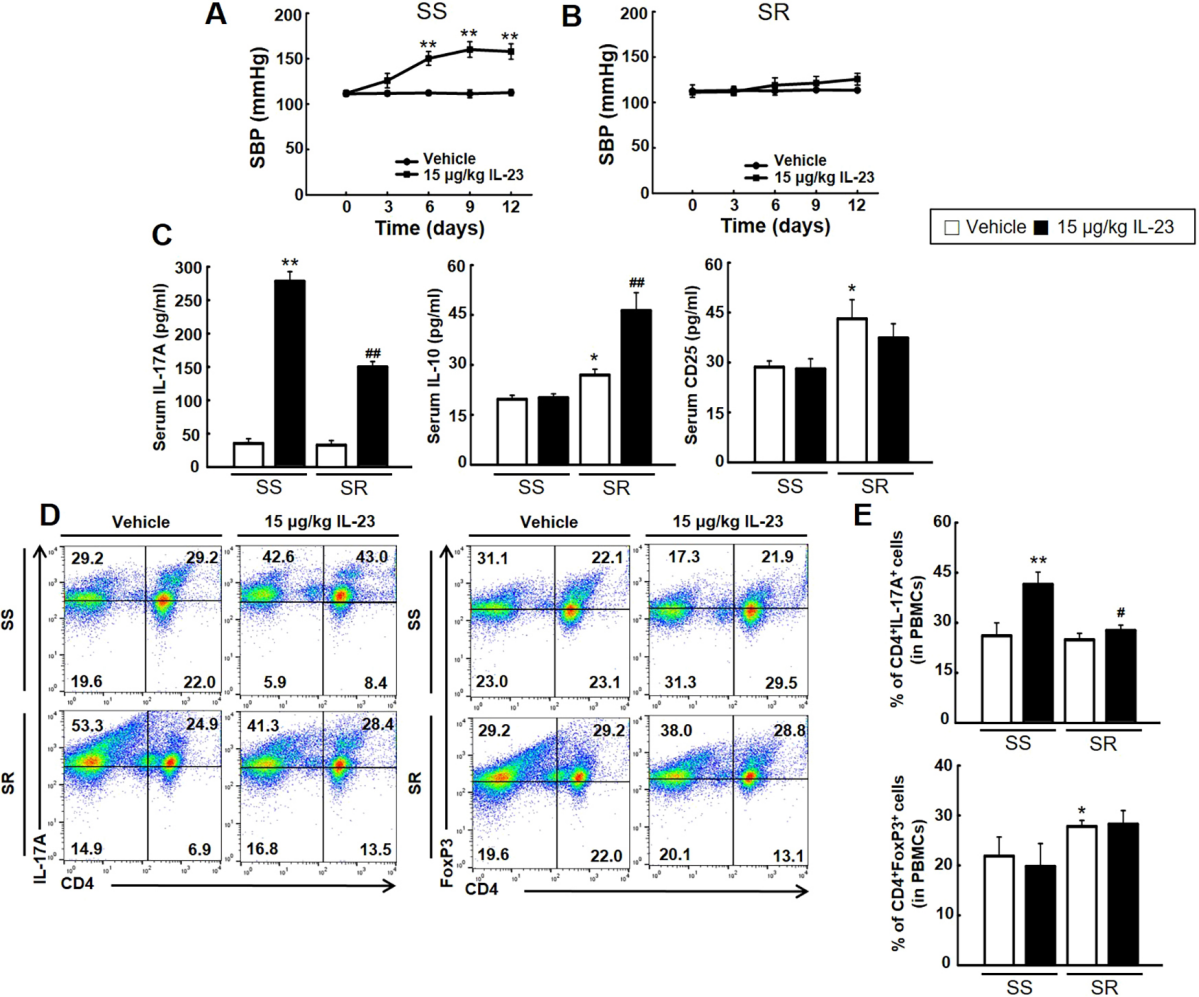
**Effect of IL-23 injection on the blood pressure of SS and SR rats.** Vehicle (PBS containing 0.1% bovine serum albumin) with or without 15 μg/kg recombinant IL-23 was injected into SS and SR rats for 12 days once every 2 or 3 days. (A,B) SBP was measured for 12 days once every 3 days. Injection of IL-23 significantly increased the blood pressure of SS but not SR rats. [Student's *t*-tests were performed for the analysis of significant differences between the two groups. Data shown are the mean±s.e.m. of six independent experiments (**P<0.01 versus the SS rat control group).] (C) The levels of serum IL-17A, IL-10 and CD25 were detected via ELISA. Injection of 15 μg/kg IL-23 increased the level of IL-17A more in SS than in SR rats. [Student's *t*-tests were performed for the analysis of significant differences between the two groups. Data shown are the mean±s.e.m. of six independent experiments (*P<0.05 and **P<0.01 versus the SS control group; ^##^*P*<0.01 versus the SR control group).] (D,E) Representatives (processed by FlowJo v10.0) (D) and densitometry (E) of flow cytometry conducted on PBMCs from SS and SR rats after completion of the injection. Injection of IL-23 increased the population of Th17 lymphocytes (CD4^+^IL-17A^+^) but not Treg lymphocytes (CD4^+^FOXP3^+^) in the PBMCs of SS rats. [Student's *t*-tests were performed for the analysis of significant differences between the two groups. Data shown are the mean±s.e.m. of six independent experiments (*P<0.05 and **P<0.01 versus the SS control group; ^#^*P*<0.05 versus the SR control group).]

IL-23 plays a pivotal role in the establishment and activation of pathogenic Th17 lymphocytes in several immune diseases (McKenzie et al., 2006). We injected recombinant IL-23 protein into SS and SR rats to investigate whether IL-23 plays a role in inducing hypertension through activation of pathogenic Th17 lymphocytes. Injection of IL-23 notably increased the blood pressure of SS but not SR rats (Fig. 7A,B). After IL-23 injection, we examined the levels of blood cytokines and secretory cell surface marker using ELISA. Injection of IL-23 increased the level of serum IL-17A much more in SS than in SR rats, and the level of serum IL-10 only in SR rats, with little change in the levels of CD25 in either group (Fig. 7D). Injection of IL-23 increased the population of CD4^+^IL-17A^+^ (Th17) cells in PBMCs and spleen T lymphocytes more in SS rats than in SR rats. However, IL-23 injection into SS and SR rats had little effect on the population of CD4^+^FOXP3^+^ (Treg) cells (Fig. 7D,E; Fig. S6).

## DISCUSSION

In the present study, we demonstrated that activation of pathogenic Th17 lymphocytes induces hypertension after high-fructose intake in SS rats but not SR rats. Furthermore, the transfer of Th17 lymphocytes isolated from donor SS rats on 20% fructose induced hypertension in recipient SS but not recipient SR rats. Meanwhile, the injection of IL-23 induced hypertension via activation of pathogenic Th17 lymphocytes in SS but not SR rats.

High-fructose intake for 4 weeks significantly increased the blood pressure of SS but not SR rats (Fig. 1). Previous studies showed that high-fructose intake induces insulin resistance, glucose intolerance and vascular resistance (Baena et al., 2016; Tran et al., 2009). High-salt diet induced whole-body and muscle insulin resistance in SS rats but not SR rats (Ogihara et al., 2002; Shehata, 2008). Our data showed that high-fructose intake caused glucose intolerance in SS rats but not SR rats (Fig. S1). There was a report that vascular insulin resistance in spontaneously hypertensive rat contributes to elevated peripheral vascular resistance and subsequent hypertension (Xing et al., 2013). However, high-fructose intake had little effect on vascular reactivity in the present study (Fig. S2).

The same trends were observed with blood pressure, level of serum IL-17A and the population of CD4^+^IL-17A^+^ (Th17) cells, which were increased in SS rats but not SR rats (Fig. 2). Similarly, high-fructose intake strongly increased the mRNA expression of pathogenic Th17 lymphocyte-related genes (*Il-17a*, *Il-17ra*, *Il-23r* and *RORγt*) in the spleen of SS rats (Fig. 3A). By contrast, the base levels of serum IL-10 and CD25 were higher in SR rats compared to SS rats and were further increased by high-fructose intake. The base population of CD4^+^FOXP3^+^ (Treg) cells was also higher in SR rats compared to SS rats (Fig. 2). In addition, high-fructose intake increased the mRNA expression of suppressive Treg lymphocyte-related genes (*Il-10*, *Cd25*, *Foxp3* and *Tgfβ*) in the spleen of SR rats (Fig. 3B). Previous work focused on the role of infiltrating T cells in the kidney during the development of salt-sensitive hypertension and renal disease in SS rats (Rudemiller et al., 2014). In our experiments, high-fructose intake significantly increased the mRNA expression of Th17-related factors and of Treg lymphocyte-related genes in the kidneys of SS and SR rats, respectively (Fig. S4). Based on our results, the immune mechanisms related to the regulation of blood pressure after high-fructose intake are quite different between SS and SR rats. SS rats show pathogenic responses after high-fructose intake, whereas SR rats show protective responses.

Some interesting results were observed due to the effects of high-fructose intake on changes in the level of serum IL-17A and the population of CD4^+^IL-17A^+^ (Th17) cells in SS rats, and we thought that these could be related. To verify the relationship, we tried an adoptive cell transfer experiment (Fig. 6A). Two types of CD4^+^IL-17A^+^ (Th17) cells were isolated from SS rats that had been maintained on either 20% fructose solution or tap water for 4 weeks, and the cells were subsequently injected into recipient SS and SR rats. From the results, CD4^+^IL-17A^+^ (Th17) cells isolated from SS rats maintained on 20% fructose solution induced hypertension in recipient SS but not SR rats (Fig. 6B), whereas CD4^+^IL-17A^+^ (Th17) cells isolated from SS rats maintained on tap water did not affect blood pressure in either recipient group (Fig. 6C). In particular, we observed a significant change in the level of serum IL-17A and the population of CD4^+^IL-17A^+^ (Th17) cells after the adoptive transfer of Th17 cells (from SS rats on 20% fructose solution) into recipient SS rats (Fig. 6D-F). These novel findings show that high-fructose intake induces the conversion of non-activated Th17 cells into activated pathogenic Th17 cells, which play a pivotal role in hypertension, and that the recipient environment is as important as the donor environment for the development of hypertension.

SGK1, also known as salt-sensing kinase, plays a major role in the cellular stress response and the downstream activation of ion channels (Lang and Shumilina, 2013). Several studies have shown that SGK1 is crucial for regulating IL-23R expression and stabilizing the pathogenic Th17 cell phenotype by phosphorylation and cytosolic translocation of FOXO1, a direct repressor of IL-23R expression in high-salt-induced inflammation and angiotensin II-induced hypertension (Kleinewietfeld et al., 2013; Wu et al., 2018). FOXP3 (a master transcription factor for the Treg lymphocyte) plays a critical role in regulating homeostasis and the activation of Treg cells. FOXO1/3 induce the stabilization of FOXP3 in the nucleus, whereas the phosphorylation of FOXO1/3 by SGK1 causes their translocation into the cytosol for degradation (Hernandez et al., 2015; Ouyang et al., 2010). As per our study results, high-fructose intake upregulates not only the mRNA and protein expression of SGK1 but also its phosphorylation level (the activity of SGK1) in SS rats only. SGK1 activation induces pathogenic Th17 cells in SS rats on high-fructose intake. However, the base activity of SGK1 was lower in SR rats than in SS rats (Fig. 4). In SR rats, high-fructose intake increased the expression of FOXO1/3 but did not affect their phosphorylation (Fig. 5). Under the high-fructose intake condition, lower SGK1 activity allows higher nuclear FOXO1/3 expression and maintained activation of Treg cells in SR rats by stabilization of FOXP3 in the nucleus. It is evident that high-fructose intake activates SGK1 in the same way that high-salt intake does during the development of hypertension in SS rats.

IL-23 is a member of the IL-12 family and is mainly secreted by mononuclear macrophages and dendritic cells. IL-23R is a well-known erythropoietin receptor family member and is expressed in activated or memory T cells. Upon binding to IL-23R, IL-23 activates downstream signaling and promotes the production of pro-inflammatory effectors such as IL-17A, IL-23R and RORγt, which are important factors in Th17 cell proliferation and activation (Harwani et al., 2016; Van Beusecum et al., 2019). The IL-23–Th17 signaling axis is related to several inflammatory diseases, such as experimental autoimmune encephalomyelitis, psoriasis and inflammatory bowel disease. A few studies have demonstrated the role of the IL-23–Th17 signaling axis in cardiovascular diseases (Krebs et al., 2014; Madhur et al., 2010). In our study, high-fructose intake increased the mRNA expression of *Il-23r* and downstream signaling effector molecules in SS rats (Fig. 3A). In view of these results, it seems that high-fructose intake affects IL-23R expression and modulates its downstream SGK1 signals. IL-23 acts as a stimulator and regulator for activating pathogenic Th17 lymphocytes, which induces hypertension only in SS rats through increasing the population of CD4^+^IL-17A^+^ (Th17) cells and the level of serum IL-17A (Fig. 7).

**Fig. 8.**
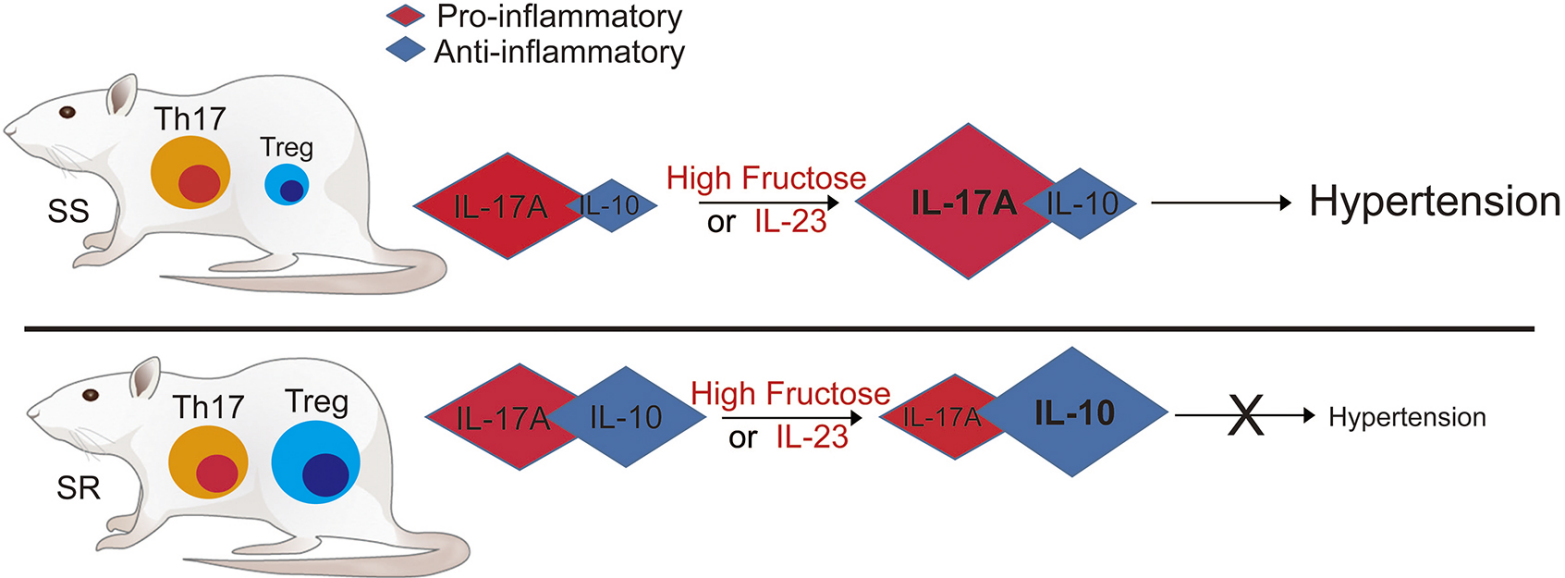
**Summary of the current** **study.** High-fructose intake or injection of IL-23 differentially affected SS rats and SR rats. In SS rats, high-fructose intake or injection of IL-23 induced hypertension by causing the production of a large amount of pro-inflammatory cytokine IL-17A secreted by activated pathogenic Th17 lymphocytes. However, SR rats were protected from hypertension following high-fructose intake or injection of IL-23 by producing a large amount of anti-inflammatory cytokine IL-10, which is secreted by Treg lymphocytes. Therefore, high-fructose intake or injection of IL-23 induces hypertension via the immunologic activation of pathogenic Th17 lymphocytes in SS but not SR rats.

In summary, this study has revealed the mechanism by which activation of pathogenic Th17 lymphocytes induces hypertension after high-fructose intake in SS rats but not SR rats. High-fructose intake differentially affects SS and SR rats. In SS rats, it induces hypertension by increasing the population of CD4^+^IL-17A^+^ (Th17) cells, which secrete pro-inflammatory cytokine IL-17A. However, SR rats are protected from hypertension following high-fructose intake by producing a large amount of anti-inflammatory cytokine IL-10, which is secreted by Treg lymphocytes (Fig. 8). In addition, the IL-23–Th17 signaling axis, which includes the IL-23R–SGK1–FOXO1/3 pathway, induces hypertension through activation of pathogenic Th17 cells in SS rats but not SR rats. Finally, we suggest that the regulation of both Th17 and Treg lymphocytes could become a novel strategy to regulate hypertension after high-fructose intake.

## MATERIALS AND METHODS

### Animals

The investigation was conducted in accordance with the National Institutes of Health Guide for the Care and Use of Laboratory Animals and was approved by the Institutional Review Board of Kyungpook National University (Approval No. 2018-0101). Every effort was made to minimize both the number of animals used and their suffering. Seven-week-old male Dahl salt-sensitive rats [DIS/EisSlc (Dahl-Iwai S)] and salt-resistant rats [DIR/EisSlc (Dahl-Iwai R)] were purchased from Japan SLC, Inc. The rats were allowed free access to a chow diet containing 0.4% NaCl (SAFE, Paris, France) to acclimate for a week before being randomly assigned to either tap water (control, *n*=6) or 20% fructose solution (fructose, *n*=6) groups for a period of 4 weeks (fructose was purchased from Millipore Billerica, MA, USA). Both fructose solution and tap water were changed every 48 h, and the animals were given free access to drinking water. Rats were anesthetized with sodium pentobarbital (50 mg/kg intraperitoneally) for sacrifice, and their tissues were frozen in liquid nitrogen and stored at −80°C until they were studied further.

### Blood pressure measurements

The SBP of the rats was measured by the tail-cuff method. Rats were preheated on a hotplate at 35°C for 10 min and then placed in plastic restrainers. A cuff with a pneumatic pulse sensor was attached to the tail. Blood pressure values were recorded on a CODA system (Kent Scientific Corporation, Torrington, CT, USA) with heating and were averaged from at least ten consecutive readings obtained from each rat.

### GTT

After high-fructose intake for 4 weeks, a GTT was performed. Rats were fasted 16 h before the GTT experiment. Fasting glucose level was determined with an ACCU-CHEK Performa (Roche, Berlin, Germany). Glucose (20% solution, 2 g/kg) was injected intraperitoneally and then blood glucose level was checked at 0, 30, 60 and 120 min.

### Aorta preparation and tension recording

After the thoracic aorta was excised, it was immediately immersed in modified Krebs solution of the following composition: 115.0 mmol/l NaCl, 4.7 mmol/l KCl, 2.5 mmol/l CaCl_2_, 1.2 mmol/l MgCl_2_, 25.0 mmol/l NaHCO_3_, 1.2 mmol/l KH_2_PO_4_ and 10.0 mmol/l glucose. The aorta was cleaned of all adherent connective tissue using wet filter paper, soaked in Krebs solution and cut into four ring segments (4.0 mm long), as described previously (Lee et al., 2016). Some rings were denuded of endothelium by gently rubbing the internal surface with the edge of a forceps. Two stainless steel triangles were inserted through each vessel ring. Each aortic ring was suspended in a water-jacketed organ bath (20 ml) maintained at 37°C and aerated with a mixture of 95% O_2_ and 5% CO_2_. One triangle was anchored to a stationary support and the other was connected to an isometric force transducer (FT03C; Grass Instruments, Quincy, MA, USA). Rings were stretched to an optimal resting tension of 2.0 g, which was maintained throughout the experiment. Each ring was equilibrated in the organ bath solution for 90 min before the experiment involving the contractile response to the addition of 50 mmol/l of KCl was conducted. Isometric responses were recorded using a computerized data acquisition system (PowerLab/8SP; AD Instruments, Castle Hill, NSW, Australia). Cumulative contractile responses were obtained after serial addition of phenylephrine (PE). Cumulative vasorelaxant responses were obtained in aortic rings with or without endothelium by serial addition of acetylcholine or sodium nitroprusside, respectively.

### Measurement of serum IL-17A, IL-10 and CD25 levels

After sacrifice, serum from the rats was collected and stored at −80°C before analysis. Serum IL-17A, IL-10 and CD25 were analyzed using ELISA, which was performed according to the manufacturer's instructions with ELISA kits [IL17A (BMS635; Thermo Fisher Scientific, Waltham, MA, USA), IL-10 (R1000; R&D Systems, Minneapolis, MN, USA), CD25 (E-EL-R0899; Elabscience Biotechnology, Houston, TX, USA)]. The optical density value at 450 nm was measured. The concentrations of IL-17A, IL-10 and CD25 were calculated according to the standard curves.

### Flow cytometry

Single-cell suspensions of spleen were generated as has been described previously (Taylor et al., 2018). Blood was obtained from SS and SR rats, which had taken 20% fructose or tap water for 4 weeks. PBMCs were separated by Ficoll-Paque PLUS gradient centrifugation (GE Healthcare, Chicago, IL, USA). Briefly, phenotypic and intracellular analyses were performed by incubating cells with antibodies for T cell surface markers – fluorescein isothiocyanate (FITC)-conjugated mouse anti-rat CD3 (1:100; clone G4.18, #554832; BD Biosciences, Franklin Lakes, NJ, USA) and PE-conjugated mouse anti-rat CD4 (1:100; clone OX-35, #554838; BD Biosciences) – for 30 min on ice in the dark. After washing, the cells were fixed and permeabilized using fix/perm concentrate (BD Biosciences) before incubation with antibodies for intracellular staining of APC rat anti-rat IL-17A (1:100; clone eBio17B7, #17-7177-81; Thermo Fisher Scientific) to identify Th17 cells and PerCP-cyanine5.5 rat anti-rat FOXP3 (1:100; clone FJK-16s, #14-5773-82; Thermo Fisher Scientific) to identify Treg cells. Cells were then washed and run through a four-color flow cytometer (FACSCalibur; BD Biosciences) and data were collected using CellQuest or FlowJo v10.0 software. An example of the flow cytometry gating strategy used is shown in Fig. S3.

### Isolation of Th17 lymphocytes and adoptive cell transfer

Blood was obtained from SS rats that had been maintained on 20% fructose (*n*=4) or tap water (*n*=4) for 4 weeks. PBMCs were separated by Ficoll-Paque PLUS gradient centrifugation (GE Healthcare). Th17 lymphocytes were then sorted by high-speed flow cytometry (FACS Aria; BD Biosciences) to a purity of >90%, as verified by post-sort analysis using CD3-FITC (1:100; BD Biosciences), CD4-PE (1:100; BD Biosciences) and IL-17A-APC (1:100; Thermo Fisher Scientific). Th17 lymphocytes were isolated with the following parameters: CD3^+^CD4^+^IL-17A^+^. The sorting strategy for the Th17 lymphocytes is shown in Fig. S5. Before adoptive cell transfer, the SBP of the rats (0 day) was measured by the tail-cuff method. To perform adoptive cell transfer, recipient rats were anesthetized with ketamine (150 mg/kg; Yuhan, Seoul, South Korea) and xylazine (18 mg/kg; Bayer, Seoul, South Korea). Recipient SS (12-week-old male, *n*=6) and SR (12-week-old male, *n*=6) rats received 300 μl of 1×10^5^ Th17 lymphocytes through intraperitoneal injections (using a 1.0 ml syringe with a 26-gauge needle) from donor SS rats that had either been maintained on 20% fructose solution or tap water for 4 weeks. After adoptive Th17 cell transfer, recipient rats were allowed to stabilize 1 day before measuring blood pressure for 2 consecutive days. During the experimental periods, the rats were fed a chow diet [containing 0.4% NaCl (SAFE)] and provided with normal tap water. On the fourth day of the experiment, rats were sacrificed under anesthesia with sodium pentobarbital (50 mg/kg intraperitoneally). After sacrifice, the rat tissues and blood were collected for further studies.

### Injection of recombinant IL-23 protein into SS and SR rats

To prepare material for IL-23 injection experiments, recombinant rat IL-23 protein (R&D Systems) was dissolved in vehicle (PBS containing 0.1% bovine serum albumin). For the IL-23 injection experiments, vehicle (200 μl) or 15 μg/kg recombinant rat IL-23 protein in vehicle (200 μl) was subcutaneously injected into 8-week-old male SS and SR rats for 12 days once every 2 or 3 days.

### qRT-PCR

Tissues (∼100 mg) were homogenized in liquid nitrogen with a glass homogenizer. Total RNA was extracted using QIAzol^®^ Lysis Reagent (QIAGEN Science, Germantown, MD, USA) according to the manufacturer's instructions. RNA was converted into complementary DNA (cDNA) using a RevertAid™ First-Strand cDNA Synthesis Kit (Thermo Fisher Scientific) according to the manufacturer's instructions. Next, qRT-PCR was conducted using an ABI Prism 7500 sequence detection system (Applied Biosystems, Foster City, CA, USA). Twenty microliters of reaction volume contained 10 μl SYBR Green Master Mix (New England Biolabs, Ipswich, MA, USA), 4 μl cDNA and 200 nmol/l primer set. The PCR reactions were conducted as follows: 2 min at 50°C, 10 min at 95°C and 40 cycles at 95°C for 15 s, followed by 1 min at 60°C. The relative expression levels were determined as the Δcycle threshold (ΔCt). All primer sets used in the present study are shown in Table S1.

### Western blot analysis

Protein-matched samples (Bradford assay) were electrophorized [SDS-polyacrylamide gel electrophoresis (PAGE)] and then transferred onto nitrocellulose membranes. These were blocked with 5% skimmed milk in TBST (25 mmol/l Tris base, 150 mmol/l NaCl and 0.1% Tween 20) for 2 h at room temperature and then incubated with primary antibody at 4°C overnight. Following incubation, the membranes were treated with a secondary antibody (1:5000) at room temperature for 1 h, followed by three washes (10 min each) with TBST. Target proteins were detected with enhanced chemiluminescence (ECL) plus detection reagents (Amersham, Pittsburgh, PA, USA). Expression levels were quantified by optical densitometry using ImageJ software (http://rsbweb.nih.gov). Western blotting was performed with the following antibodies: anti-p-SGK1/SGK1 (1:1000; ab55281/ab59337; Abcam, Cambridge, UK), anti-p-FOXO1 (1:1000; #9464; Cell Signaling Technology, MA, USA), anti-p-FOXO3 (1:1000; ab47285; Abcam), anti-FOXO1/FOXO3 (1:1000; ab39670/ab12161; Abcam), anti-lamin B (1:2000; sc-6217; Santa Cruz Biotechnology, Santa Cruz, CA, USA) and anti-glyceraldehyde 3-phosphate dehydrogenase (GAPDH; 1:2000; sc-365062; Santa Cruz Biotechnology).

### Statistics

SPSS software (release 25.0; SPSS Inc., Chicago, IL, USA) was used to analyze the data. The Kruskal–Wallis test and one-way ANOVA followed by Tukey's HSD post hoc tests were applied to compare multiple data. Student's *t*-tests were performed for the analysis of significant differences between two groups. Results were expressed as mean±s.e.m. and *P*<0.05 was considered significant.

## Supplementary Material

Supplementary information
