# Supplementary material for: Activated pathogenic Th17 lymphocytes induce hypertension following high-fructose intake in Dahl salt-sensitive but not Dahl salt-resistant rats
Source: Dis Model Mech. 2020 May 27;13(5):dmm044107. doi: 10.1242/dmm.044107 (PMC7272342; doi:10.1242/dmm.044107)
Supplement: Supplementary information [file dmm-13-044107-s1.pdf]

Table S1. Primers for qRT-PCR

Gene (Accession No.)	Primer sequence (5' to 3')	
IL-17A	F:	GAAGGTCAACCTGAAAGTCC
(NM_001106897.1)	R:	GGATATCTATCAGGGTCCTC
IL-17RA	F:	TCTTAGTGCCTGGCTGCGAG
(NM_ 001107883.2)	R:	CACAGGGTGAAGTCCACTCG
IL-23R	F:	CCAGTAGGCAGACAACCCTA
(NM_ 001191750.1)	R:	AGCTGTGAGAGTTCCTGTAG
RORγt	F:	ACTCAGCTGAAGACGCTGAG
(NM_ 001015011.2)	R:	GCACACCTTACTGAGAGACC
IL-10	F:	GCATCTACTGGACTGCAGGA
(NM_ 012854.2)	R:	GGAGAGAGGTACAAACGAGG
CD25	F:	CCAAATCCTTGCTGGTCTGT
(NM_013163.1)	R:	CTCAGAGCCTAGACGGATGG
TGF-β	F:	TGGACCGCAACAACGCAATC
(NM_021578.2)	R:	TTCTGGCACTGCTTCCCGAA
FOXP3	F:	ACA AGGATCCTACCCACTGC
(NM_ 006256731.3)	R:	ATGCAGTTTAGCCCTTTGCT
SGK1	F:	CGTATTTCCCATGTGTGCAG
(NM_001193568.1)	R:	GTCACTGCAAGACCGTTTCA
FOXO1	F:	GTGAACACCATGCCTCACAC
(NM_001191846.2)	R:	CACAGTCCAAGCGCTCAATA
FOXO3	F:	GGGGAGTTTGGTCAATCAGA
(NM_001106395.1)	R:	TTTGCATAGACTGGCTGACG

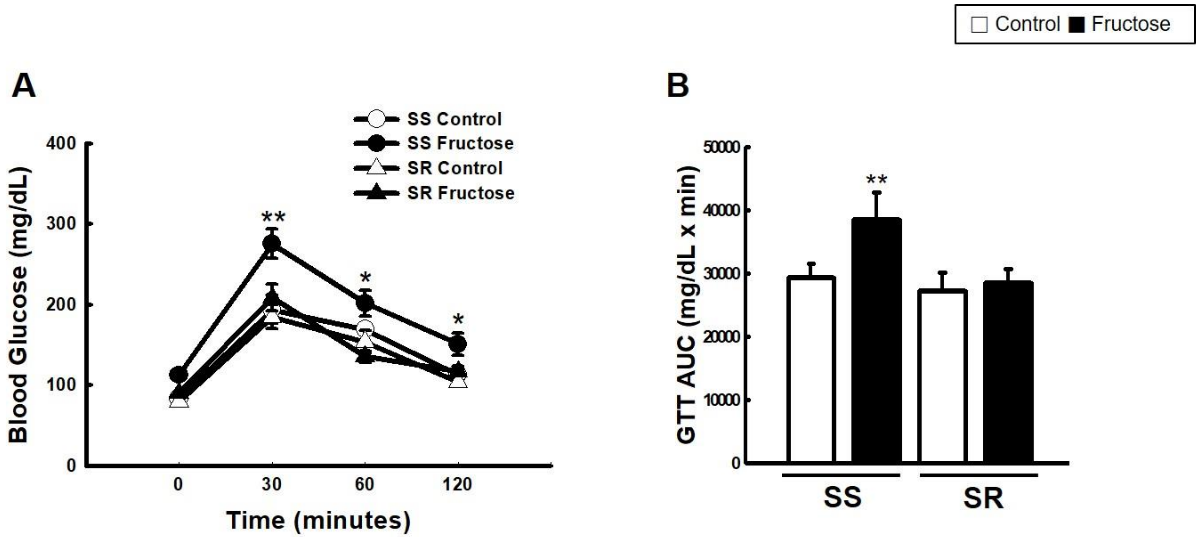

<u>Figure S1</u>. Effect of high-fructose intake on glucose metabolism. (A) Blood glucose was measured at 0, 30, 60 and 120 minutes after intraperitoneal injection of glucose in SS and SR rats fed tap water (control, n = 4) or 20% fructose solution for four weeks. High-fructose intake significantly increased the blood glucose level of SS rats at 30 minutes after intraperitoneal injection of glucose. (B) Glucose tolerance test were assessed as area under the curve (AUC). High-fructose intake significantly increased the AUC value in SS rats. (Data shown are the mean ± SEM of six independent experiments. **$p < 0.01$ vs. the SS rat control group)

Disease Models & Mechanisms • Supplementary information

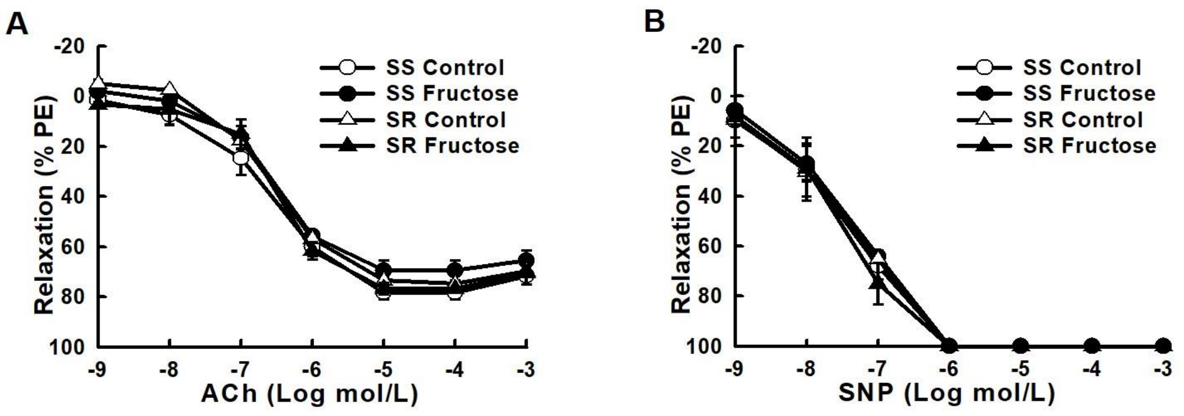

Figure S2. Effect of high-fructose intake on vascular relaxation. SS and SR rats were offered tap water or 20% fructose solution for four weeks. Relaxation curves show the relaxant responses to the cumulative addition of acetylcholine (ACh) and sodium nitroprusside (SNP) in the aortic rings with (A) and without (B) endothelium, respectively. High-fructose intake did not affect the relaxation. Developed relaxation is expressed as a percentage to the maximal tension to phenylephrine (PE) .

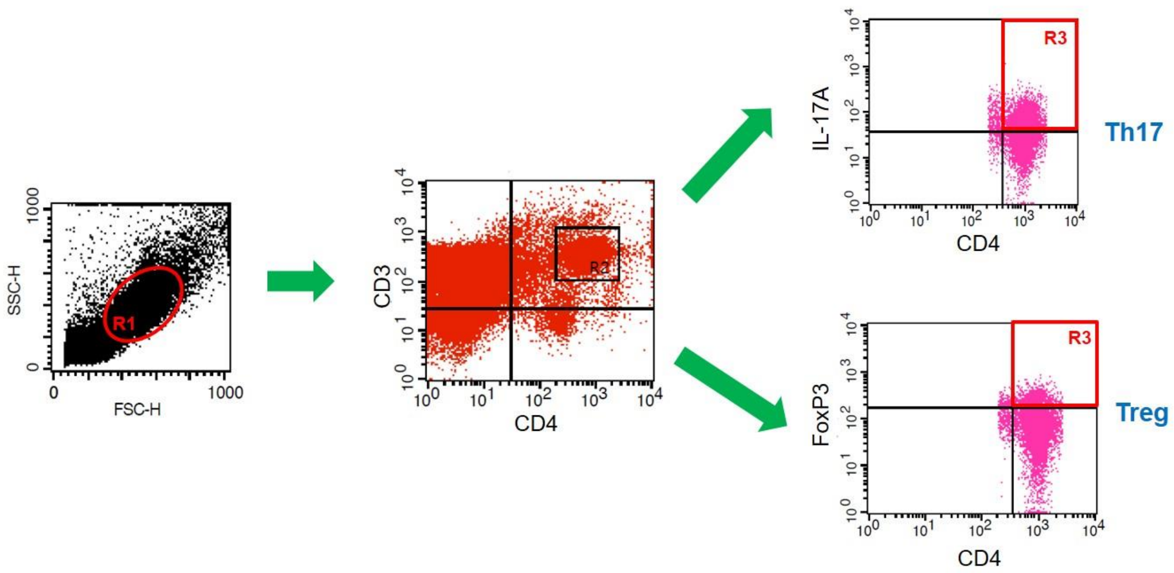

<u>Figure S3</u>. Flow cytometry gating strategy. The selection of cells based on forward scatter (FSC) and side scatter (SSC). T cells were analyzed based on the expression of CD3 and/or CD4. $CD3^{+}CD4^{+}$ T lymphocytes were further gated for expression of IL-17A for Th17 lymphocytes and for expression of FoxP3 for Treg lymphocytes.

Disease Models & Mechanisms • Supplementary information

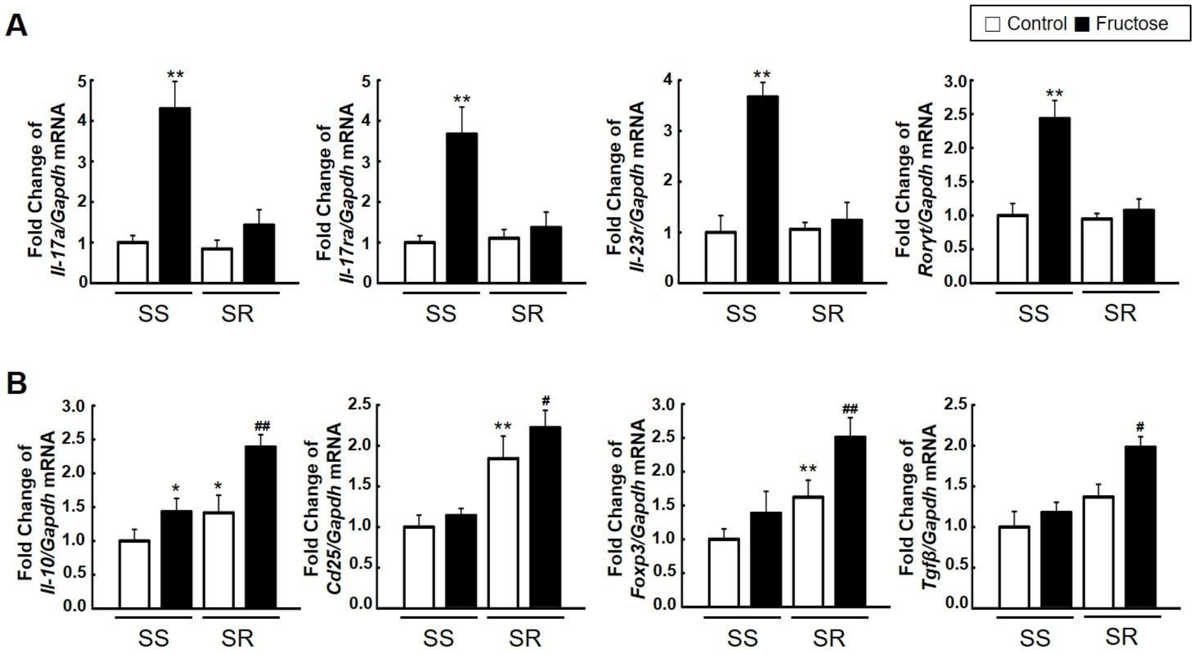

<u>Figure S4</u>. Effects of high fructose intake on the expression of Th17 and Treg lymphocytes-related genes in kidneys of SS and SR rats. (A) The mRNA expression levels of Th17 lymphocyte-related genes *Il-17a*, Il-17 receptor a (*Il-17ra*), Il-23 receptor (*Il-23r*) and retinoid-related orphan receptor *γt* (*RORγt*) in the kidneys of SS and SR rats were measured by quantitative real-time PCR (qRT-PCR). (Student's t-tests were performed for the analysis of significant differences between the two groups. Data shown are the mean ± SEM of six independent experiments. (**$p < 0.01$ vs. the SS rat control group; $^{\#}p < 0.05$ vs. the SR rat control group)). High fructose intake increased the mRNA expression of Th17 lymphocyte-related genes more in SS than in SR rats. (B) The mRNA expression levels of Treg lymphocyte-related genes forkhead box P3 (*Foxp3*), *Cd25*, *Il-10*, and transforming growth factor *ß* (*Tgf ß*) were measured by qRT-PCR in the kidneys of SS and SR rats. High fructose intake increased the mRNA expression of Treg lymphocyte-related genes more in SR than in SS rats. (Student's t-tests were performed for the analysis of significant differences between the two groups. Data shown are the mean ± SEM of six independent experiments. (*$p < 0.05$ and **$p < 0.01$ vs. the SS rat control group; $^{\#}p < 0.05$ and $^{\#\#}$ $p < 0.01$ vs. the SR rat control group)).

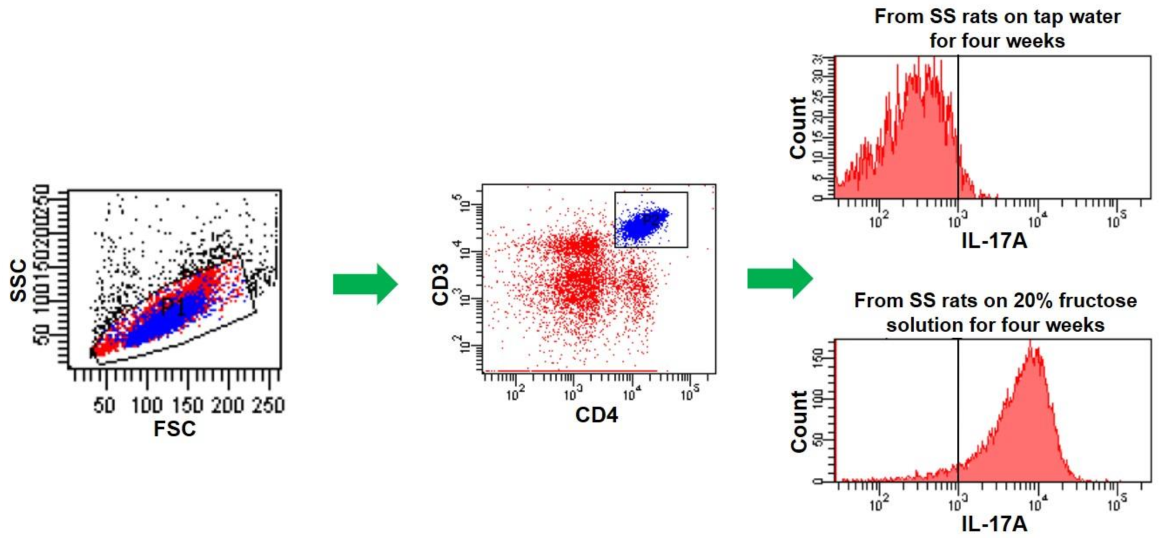

<u>Figure S5</u>. Isolation strategy of Th17 lymphocytes from peripheral blood mononuclear cells (PBMCs). The selection of cells based on forward scatter (FSC) and side scatter (SSC). T lymphocytes were selected based on the expression of both CD3 and CD4. $CD3^{+}CD4^{+}$ T lymphocytes were further gated for expression of IL-17A for Th17 lymphocytes.

Disease Models & Mechanisms • Supplementary information

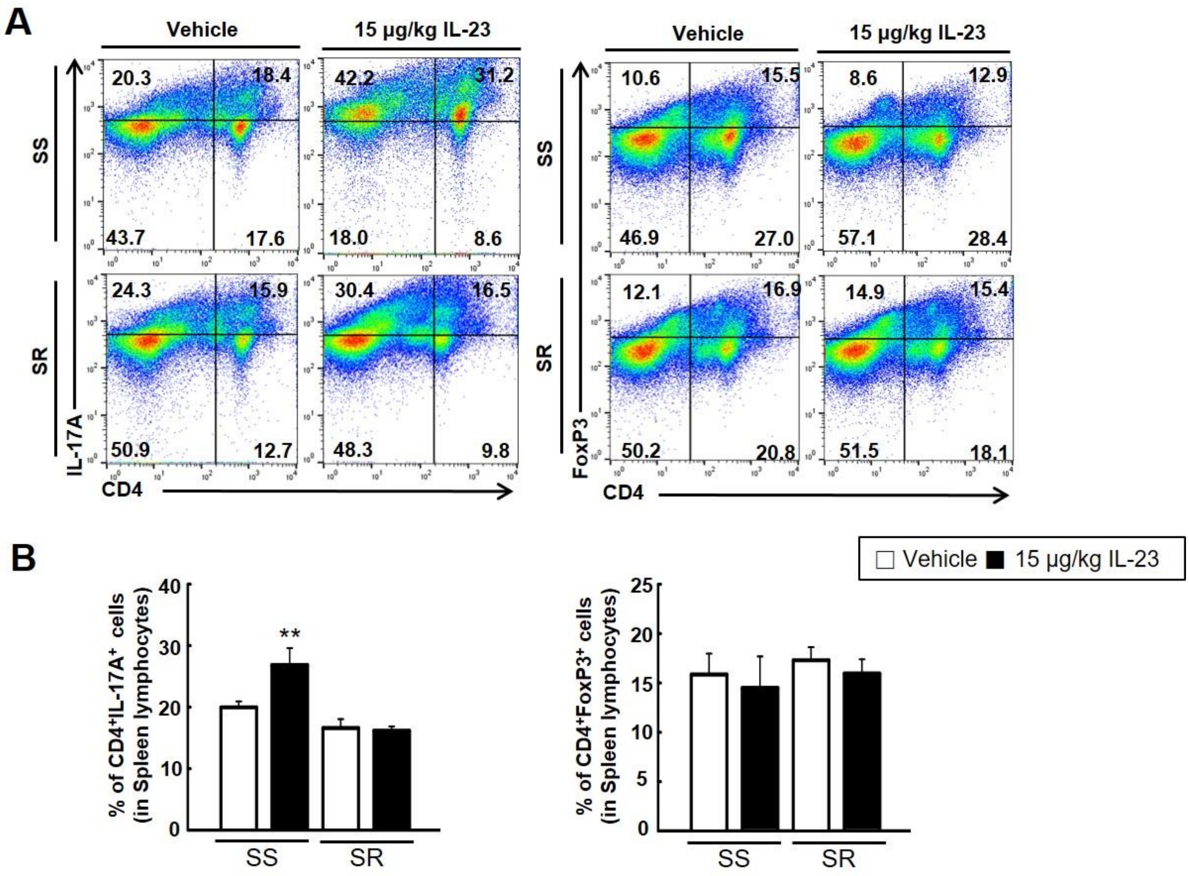

<u>Figure S6</u>. Effect of IL-23 injection on the population of Th17 lymphocytes ($CD4^{+}IL\text{-}17A^{+}$) and Treg lymphocytes ($CD4^{+}$ $FoxP3^{+}$) in the spleen lymphocytes of SS and SR rats. Injection of IL-23 increased the population of Th17 lymphocytes (CD4+IL-17A+) but not Treg lymphocytes (CD4+ FoxP3+) in the splenocytes of SS rats. (Data shown are the mean ± SEM of six independent experiments. **p < 0.01 vs. the SS rat control group)

Disease Models & Mechanisms • Supplementary information
